# Prospects of digital scientific publishing on blockchain: The concept of DAP

**DOI:** 10.12688/openreseurope.15771.2

**Published:** 2024-11-27

**Authors:** Karolj Skala, Zorislav Šojat, Josip Maričević, Davor Davidović, Viktor Bojović, Tomislav Zubčić, Branimir Kolarek, Dario Pažin, Draško Tomić, Tadej Slapnik, Mario Pecimotika

**Affiliations:** 1 Centre for Informatics and Computing, Ruđer Bošković Institute, Zagreb, 10 000, Croatia; 2 Mone Code ltd, Zagreb, 10 000, Croatia; 3 HasNnet ltd, Slovenske Konjice, Slovenia

**Keywords:** Academic publishing, blockchain, distributed ledger technology, distributed computing, smart contract, transparent peer review, reviewer success ranking, reviewer recognition, HashNet

## Abstract

**Background:**

Traditional publishing models, open access and major publishers, cannot adequately address the key challenges of academic publishing today: Speed of peer review, recognition of work and incentive mechanisms, transparency and thrust of the system.

**Methods:**

To address these challenges, the authors propose Democratisation of Academic Publishing (DAP) platform, which is based on the novel HashNET DLT platform. The DAP introduces several innovative components: tracking the activities of all participants in the peer review process using blockchain and smart contracts, the introduction of the Scholarly Wallet for holding reputation (non-fungible) and reward (fungible) tokens, the use of the Scholarly Wallet as the main interface to the DAP platform, the Virtual Editor that enables automatic discovery of the research area and invitation of reviewers, and finally the global database of evaluated reviewers, ranked by the quality of their previous work.

**Results:**

The DAP platform is in the development phase, with the design and functionalities of all modules defined. An exception is the central component of DAP, the Scholarly Wallet module, whose first prototype has already been created, tested and published. The implementation of DAP is planned for the next phase of the HorizonEurope TruBlo project and other research initiatives. The DAP platform will be connected to the publishing ecosystem: 1) as a backend system (distributed blockchain database) for existing publishing platforms and 2) as a standalone publishing platform with its own API interface.

**Conclusions:**

The authors believe that DAP has the potential to significantly improve academic peer review and knowledge dissemination. It is expected that the use of blockchain technology, the fast HashNET consensus platform and tokens for reward (fungible) and reputation/ranking (non-fungible) will lead to a more efficient and transparent way of rewarding all participants in the peer review process and ultimately advance scientific research.

## Plain language summary

The paper "Prospects of digital scientific publishing on blockchain: The concept of DAP" proposes the use of Decentralised Academic Publishing (DAP) to improve the current system of scientific publishing. DAP will use blockchain technology to make research metadata read-only, secure and more visible. It will also use scientific tokens (Ergion) to reward reviewers, editors and authors. By using blockchain technology, DAP can offer faster and more efficient publishing processes, increase accessibility and reduce the potential for fraud. DAP will be a decentralised system managed by a community of users rather than a central authority and will use smart contracts to automate processes such as peer review services and increase transparency. Overall, the authors believe that DAP has the potential to significantly improve scientific peer review and knowledge dissemination by reducing review time and making the entire peer review system more transparent and reliable.

## Introduction

From the outset of scientific research, publishers have played an important role in the process of academic publishing as carriers of new ideas, theories and knowledge within the academic community and to the wider public. Over the years, they have become an indispensable link in the chain of academic publishing, and many publishing models have been developed and attempted since then. The concept of publishing (whether we are talking about open access publishing or subscription-based publishing, for example) is not evolving in the best interests of the researchers or the public they serve, because access to research findings and data is generally restricted depending on the platform on which the article was published. Although open science publishing platforms appear to be overcoming the above obstacles, much progress is still needed, particularly in the peer review process and in recognising the work of reviewers. The large survey conducted by Publons ^1^ in 2018, in which around 12 000 researchers worldwide participated, shows that in 2013 an editor had to invite 1.9 reviewers to get a review, while this number has increased to 2.4 in 2017 and is expected to reach 3.6 by 2025. This clearly shows that the motivation of reviewers to participate in reviewing articles has decreased significantly. This is partly due to the insufficient appreciation of the reviewers’ work. Interestingly, the majority of respondents stated that their institutions should recognise their work as reviewers more explicitly. The study concludes that better recognition and new incentive mechanisms should be introduced to motivate wider participation in the peer review process.

To address these challenges, our paper proposes an approach that uses blockchain technology to change the paradigm of publishing. By integrating a system in which reviewers are not only recognised but also actively rewarded for their contributions, we aim to create a dynamic and incentive-driven environment that fosters robust peer review. With this model, we aim to strengthen the motivation of reviewers and ultimately improve the quality and accessibility of academic knowledge for the benefit of the entire research community and the general public.

### Incentives in digital publishing

The low willingness of researchers to accept peer reviews means that it becomes more difficult for editors to find reviewers who will accept and conduct peer reviews in a timely manner, leading to frustration for all those involved in the peer review process (editors, authors, reviewers). The shortage of reviewers could force editors to invite any scientist willing to write a review, even though the appointed scientist may not understand or contribute to the article but has to write something. In our opinion, one of the major drawbacks of the current peer review process is that the pools of potential reviewers from certain publishers, journals or editors are not shared with others, even if they belong to the same research area. Under the current model, the list of reviewers cannot be shared between different publishers, editors or conference chairs, so each publisher (editor) relies on its own list of potential reviewers. The same Publons survey ^1^ also shows a strong geographical (country) correlation between publishers and reviewer selection. This also shows that 96.1% of reviewers come from established regions, with the USA leading the world in both the number of publishers and the number of completed reviews. It is therefore crucial to improve diversity and inclusivity in peer review, especially involving early-career researchers, researchers from different countries and backgrounds, and women. Furthermore, to our knowledge, there is no solution to evaluate reviewers and rank them according to the quality of their work.

In recent years, publishers have made limited efforts to recognise the work of reviewers, such as Elsevier’s Reviewer Hub ^1^, which tracks reviewer activity for Elsevier’s journals. Clarivate’s Web of Science Reviewer Recognition Service ^2^ provides integration with a journal’s existing peer review management system, which then automatically tracks and recognises reviewers. Similarly, the ORCiD Peer Review Service ^3, 4^ can be integrated with editorial management submission systems. Reviewers then enter their ORCID iD and automatically receive credit for their review activity, which is visible in their ORCiD account. Although the number of publishers and journals supporting different reviewer recognition systems is small, researchers need to track their activities through different services and accounts. In addition, evaluation of the author’s work is the next important issue, as often only manuscripts published in high impact factor journals are considered for evaluation and career advancement. This creates another barrier to publishing manuscripts on open science publishing platforms and encourages further costs to what appears to be open science.

A new and attractive way to improve the quality of published work and peer review, especially for early career scientists, is to introduce open peer review ^2, 3^. Open peer review is a relatively new model that has recently become more popular. The idea is to publish the entire peer review process (reviewers’ comments, authors’ responses, editors’ decisions), including the identity of the reviewers and editors, along with the published article. By making each link of the chain publicly accountable for their contributions, this model promotes integrity, fairness, and quality in the review process. Reviewers and editors are incentivised to provide constructive feedback as their comments are accessible to all, enabling scrutiny of the decision-making process behind publication. In 2020, Nature launched a pilot project ^4^ that allows authors to decide for themselves whether to publish anonymous reviewer reports and their communications with reviewers. This pilot was triggered by a 2017 Nature survey ^5^ of reviewers, in which 62% of respondents said that publishers should offer alternative peer review models, and 51% agreed that peer review should be more transparent. Other publishers and journals have also adopted various types of open peer review processes, such as MDPI ^6^, ORE ^5^ and PLOS ^6^. The open peer review process could improve the integrity, fairness and quality of the review process as reviewers and editors are more likely to provide constructive comments as these are publicly available so that everyone can judge how and why the decision to publish was made. It is believed that the transparent (and open) peer review process is key to the future development of academic publishing. However, there are some concerns about the fully open peer review process, as it could potentially lead to retaliation against authors if negative comments are made by reviewers. The authors in 7 simulated different scenarios and compared models for confidential peer review and open peer review. The simulations show that open peer review could increase publication bias, *i.e.* the number of manuscripts that should not be published but are published due to various sociological factors ( *e.g.* reviewers’ fear of retribution, young researchers reviewing manuscripts by older scientists, *etc.*).

### Blockchain in academia

Several studies have explored the potential of blockchain technology to improve the academic publishing process and enhance peer review to overcome issues such as slow and biassed peer review processes, high publication costs, lack of transparency, limited journal scopes and resistance to change. An example of early adoption of blockchain in academic publishing is presented in 8. The authors propose the incorporation of blockchain technology into the publishing process to create a secure, decentralised and publicly accessible timestamp for each submitted manuscript. They also explore the benefits of using blockchain, such as preventing plagiarism of unpublished results by anonymous reviewers or editorial board members prior to publication, while addressing the shortcomings of existing publication systems.

In 9, the potential benefits of blockchain technologies in open science are analysed, such as transparency in editing, reviewing and publishing academic papers, managing intellectual property, establishing identity and preventing fraud. In addition, cryptocurrencies were proposed as an incentive and reward model for reviewers and editors. Although we could not find an implementation of their work, the authors proposed a list of minimal features that a smart contract should have, as well as a detailed description of workflow integration. The proposed model is that submitting an article costs a predetermined amount of tokens, which are then distributed among editors, reviewers and publishers. The authors also point out some negative aspects of such a system, *e.g.* that it encourages overly positive or negative reviews, as reviewers are only paid once and will therefore try to minimise the number of revisions, *i.e.* their effort. According to 10, blockchain has two important applications in academia: verifying the reliability of source data and enabling transparent evaluation of academic papers and publication outlets.

Another study ^11^, proposes a decentralised publishing system that uses smart contracts on the Ethereum blockchain. This system rates users’ contributions based on a reputation score and compensates users with digital assets. According to 12, 13, a major challenge in the scientific community is the limited availability of research papers on the internet, which are usually published only after the review process. This leads to a lack of transparency and a lack of information about the review process itself. As a result, the reproducibility of research is often compromised, reviewers are often not acknowledged for their contributions and much of their work goes unnoticed. The paper proposes to use blockchain technology to address these issues by providing a way to reward reviewers and other stakeholders, as well as facilitating digital rights management and storage of research results. The author emphasises the potential of blockchain to introduce new metrics beyond what is currently measured and recorded, although it remains unclear exactly how this could be achieved.

In 14, an Ethereum-based framework is proposed that uses shared governance to ensure transparency and fairness in the scholarly publishing process. The framework consists of a decentralised network of nodes representing stakeholders who participate in a consensus mechanism to validate transactions. This system offers benefits such as faster publication times, more transparency and fewer opportunities for fraud and plagiarism. In 15, a decentralised publication system for open science is proposed that uses blockchain and IPFS (InterPlanetary File System). The system is based on three main pillars: a distributed reputation system for reviewers, OA by-design, and transparent governance. In OA by-design, academic documents, from first drafts to their final versions, including peer reviews, are distributed through the use of IPFS. In 16, the authors propose an optimal OA model for scholarly publishing that has no economic barriers, appropriate incentives for authors and reviewers, and a decentralised regulatory framework. They propose a decentralised blockchain-based solution to manage scholarly communication and solve the challenges and incentive problems of traditional systems.

In 17, the author discusses various use cases of blockchain in academia, including trusted data management, secure student records, decentralised academic publishing, and digital credential verification. The author also explores the challenges and limitations of implementing blockchain technology in higher education, such as the need for a clear regulatory framework, technical expertise and the potential impact on traditional academic practises.

In 18, an open peer review system was developed that incorporates blockchain technology and recommendation tools for reviewers. This system uses Hyperledge Fabric, a private blockchain network, for improved speed, security and optimisation for specific communities. The recommendation module checks the expertise of potential reviewers and removes potential conflicts of interest. The editor can then select the best reviewer from the list of final candidates. Manuscript submission and reviewer selection take place off-chain, while smart contracts, peer review, evaluation and reward allocation are done on-chain, with a service broker acting as an intermediary between the two parts. While the presence of the service broker can make the process more complex, it also provides additional functionality to connect the on-chain part with various services such as conference submissions and research proposal evaluations.

In 19, a novel platform for scholarly publishing based on blockchain technology and the Ethereum Virtual Machine is proposed. The authors aim to address the problem of limited availability of scientific journals and frequent submission of articles to journals from fields outside the discipline. The platform also aims to increase the speed of peer reviews and reduce reviewer bias by selecting reviewers from other institutions and regions. Rejected manuscripts are forwarded to other, more suitable journals where new reviewers do not have to start from scratch as they can consult and refer to old reviews. The system remunerates authors and reviewers regularly (every six months) based on the number of citations or the speed and quality of reviews. At the same time, authors do not stress the inadequacies of such measures to encourage further work by researchers and reviewers. One of the problems is that researchers need tokens to submit their work, which is challenging for young researchers just entering the scientific field. The number of citations has been proposed as the sole or primary criterion for rewarding authors, but this approach has several potential problems. For example, researchers tend to cite papers published in prestigious journals more frequently, resulting in an unfair advantage for authors who publish in these journals.

The number of citations may also be influenced by a number of factors, such as the author’s professional network, the field of study and the language of the paper, leading to a lack of diversity in the papers cited and awarded. Some authors may try to manipulate the system by citing themselves or including excessive citations, which can artificially inflate the number of citations and make it difficult to accurately measure the impact of a paper. In addition, using the number of citations as the sole or primary criterion for rewarding authors can lead to an emphasis on quantity rather than quality and incentivise the production of low-quality or irrelevant articles that generate large numbers of citations. Citation metrics only capture the short-term impact of a paper and do not take into account the longer-term impact, which can be much more significant. By relying solely on the number of citations, authors run the risk of overlooking the significance of work that has a lasting impact on a field. Rewarding reviewers based solely on the speed of the review process can lead to a reduction in the quality of their reviews. A rushed approach can lead to reviewers overlooking crucial details or not reviewing the work thoroughly enough, which ultimately affects the quality of the review. In addition, this approach can lead to reviewers favouring shorter and simpler papers over more complex ones, resulting in the publication of low-quality or inaccurately assessed papers and a bias against certain types of research.

Other alternatives to traditional publishing platforms that aim to overcome their limitations include PubChain ^20^ and TimedChain ^21^. In 21, a blockchain-based editorial system is presented that implements all steps from submission to publication with the participation of publishers, authors, readers and other third parties ( *e.g.* reviewers). The platform uses time-based smart contracts and advanced cryptography techniques to manage transactions, control access and improve security. It also introduces a reward mechanism for publishers that takes into account their effort and quality in managing and maintaining publications and research data. The Pluto platform ^22^ aims to build a decentralised platform without a single point of control and proposes a notion of "reputation score" calculated on the basis of research contributions. ARTiFACTS ^23^ records scientific artefacts in immutable chains to facilitate the storage and sharing of research results. The system is designed to help existing repositories of research data create, share and track data. The company SciencecMatters presented EUREKA ^24^, a blockchain-based peer-to-peer platform for scientific publications. The authors introduced the EUREKA tokens as a mechanism to reward reviewers. However, many of the above research initiatives and platforms have not reached the production stage or their further development has been abandoned, as in the case of SciencecMatters-EUREKA.

### Contribution

In this paper, we present and describe our solution to overcome the above drawbacks of modern academic publishing. The Democratisation of Academic Publishing (DAP) platform presented by the authors of this paper takes advantage of all the positive aspects and benefits of the ORE platform and provides a reliable, trustworthy and efficient infrastructure for academic publishing based on blockchain technologies that aims to overcome the drawbacks of the current publishing processes. Some other problems in the academic community that can be solved with blockchain are issues of academic integrity, reproducibility and prevention of data falsification.

The main advantages and innovations of the platform DAP presented in this article are:

- Tracking and recognition of reviewers’ work using blockchain technologies and smart contracts
- Introduction of the scholarly wallet to store reviewer tokens for ranking purposes
- Global database of evaluated reviewers, shared among editors and editorial boards
- Virtual editor that automatically recognises the scientific domain.

## The architecture of the DAP platform

The Democratisation of Academic Publishing is a decentralised academic peer review platform based on blockchain technologies. The goal of DAP is to improve the quality of the peer review process, automate, enhance, and objectify the process, and facilitate academic communication by making the entire process transparent, reliable, and trustworthy. As participants in the process, the activities of reviewers, authors, and editors are tracked and stored in the DAP platform (blockchain backend) and appropriately rewarded. Decentralisation and automation are the basic ideas of DAP.

### The ecosystem of DAP

**Figure 1.**
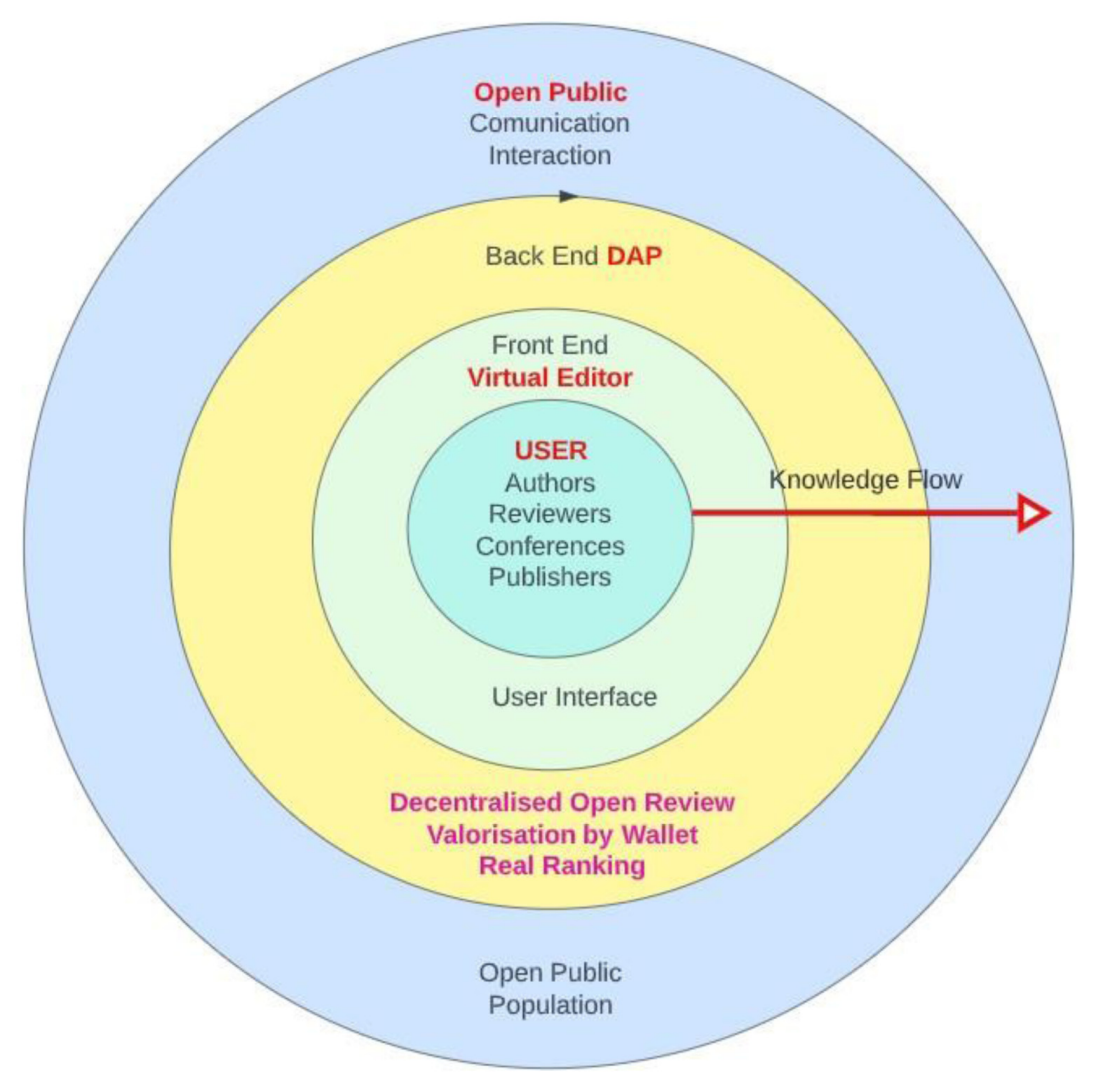
Decentralised user-centred shell model of Decentralised Academic Publishing (DAP).

**Figure 2.**
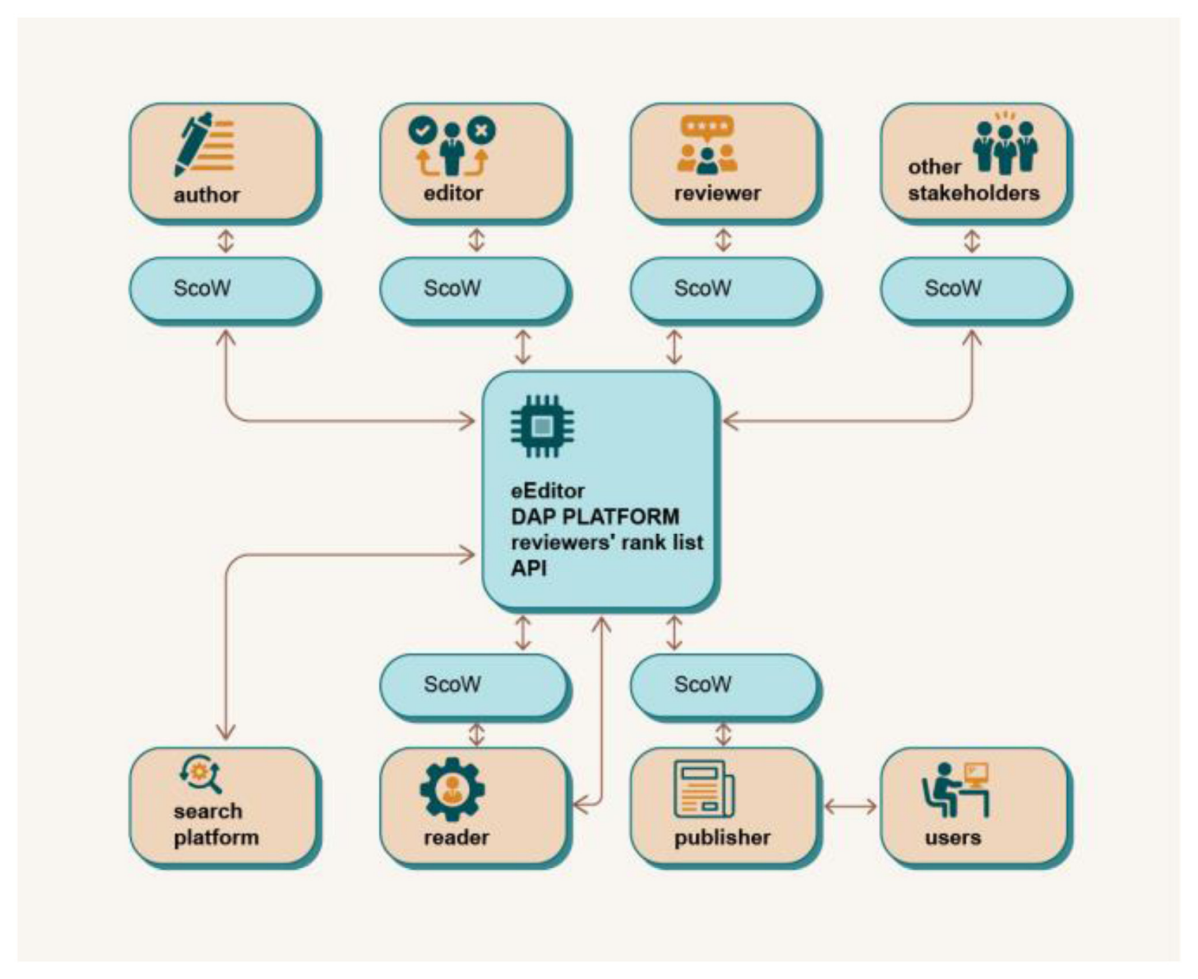
Decentralised Academic Publishing platform - a global overview.

The DAP is intended to be a global, unique platform that enables interaction and collaboration between all users of the platform, as illustrated in Figure 2. Each user has access to the platform in their specific way, depending on their role. In contrast to the current publishing system, the user is at the centre of the processes and interests of the DAP platform ( Figure 1). The decentralised nature of the DAP system prevents interference in the publication process without a strictly defined protocol, thus ensuring confidential and decent academic communication in the publication process. Participants carry out the process of manuscript submission and review through to final publication *via* the front-end interface with support from the back-end system. The system uses innovative elements such as a virtual editorial system, an open and competitive peer review process and, finally, the Scholarly Wallet (ScoW).

The DAP ecosystem consists of the infrastructure (the subsystem of the ecosystem that enables all transactions available to the users of the ecosystem) and the users. The Infrastructure Providers are the intermediaries between the infrastructure and the users and constitute a special category that has a democratic right to propose and vote at the infrastructure level (Proof of Authority, PoA), as well as all other proposal and voting rights of regular users. Each user of the ecosystem has a Scholarly Wallet (ScoW), *i.e.* an interface to the system DAP, based on identification with a private/public key (see Figure 2). Since individual users can hold several roles, a specific key combination is issued for each of their roles when they register in the system. These secondary keys are linked to the individual master key. Therefore, all rewards as well as information about users are always available from DAP (according to the visibility rules), but the specific efforts in the different roles are kept, rewarded and ranked independently. From the perspective of DAP, several main roles are identified: Author, Reviewer, Editor, Translator, Proofreader & Copywriter, Publisher, Reader, Infrastructure Provider, Academic Collective and Outsider.

Another feature of DAP, which differentiates it from other similar solutions, is that it is a global and unique platform that is not tailored to or tied to a specific publisher or user. DAP supports multiple publishers simultaneously, improves knowledge sharing and the list of reviewers across the scientific community, and breaks down barriers between publishers. Each role accesses the platform through its specific application programming interface (API) ( Figure 2). User activities and interactions with the platform are tracked on the blockchain backend and identified with a specific user’s private/public key stored in their ScoW. This solution opens up the possibility of creating a global list of reviewers ranked by the quality of their previous work in the review process. This list can be shared between publishers and editors, providing a larger pool of high-quality reviewers with expertise in a particular research area. Furthermore, using the blockchain on a global scale to track and rank users’ activities significantly reduces the fragmentation of knowledge, reviewers and editors between different publishers and increases the geographical diversification of participants in the peer review process.

### Use cases

**Figure 3.**
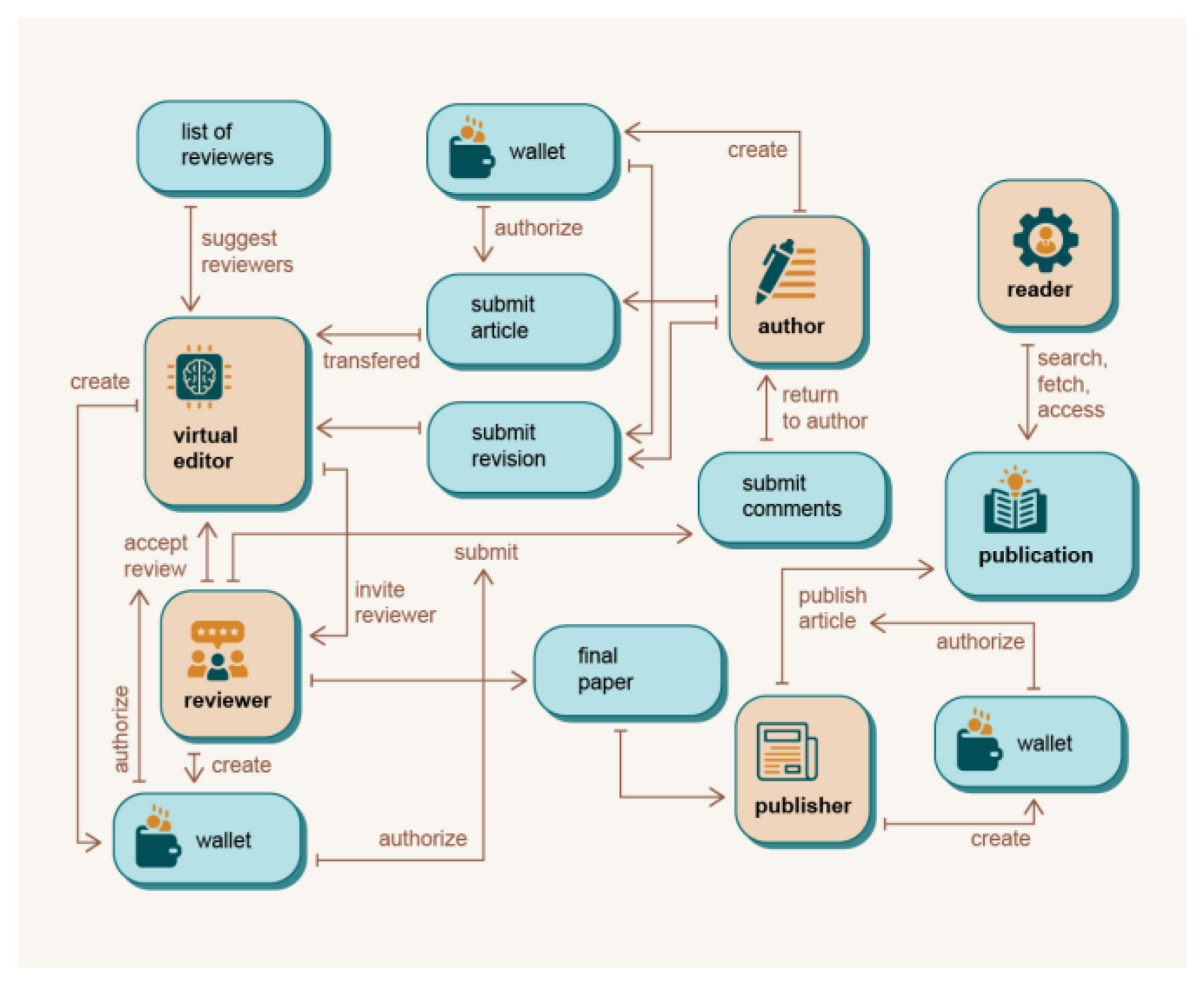
Use case diagrams for different user roles.

A general overview of how different user roles interact with the DAP is given in Figure 3. The figure describes the use cases of the five most important user roles: Author, Reviewer, Editor, Publisher and Reader.

Participants access the DAP platform *via* different interfaces, depending on their role, *via* web-based portals or *via* the existing web-based user interfaces of publishers (journals, conferences, *etc.*). These external platforms interact with the DAP platform *via* the DAP API. When the author submits an article *via* the publisher’s user interface (UI) or DAP graphical user interface (GUI) (or even the publishing platform), the metadata of the article is transmitted to the virtual editor and stored in the blockchain. The virtual editor accesses the article *via* a provided URL. The information stored in the metadata includes abstract, authors, institutions, titles, and keywords. The list of stored metadata information could be expanded in the future as needed. Using this data, the virtual editor can determine the correct section in which to include the article and attach the list of articles in that section that are available to review candidates. When review candidates select an article, they have a soft deadline and a hard deadline within which they must submit a review report. The rest of the process is the same as any other review system, except that DAP uses blockchain infrastructure to reward authors, reviewers, and other users of the system for their work in the publishing process. The entire peer review process is extracted from the blockchain and analysed using relational databases. The DAP platform can then automatically assess the quality of the reports and the reviewers based on various benchmarks yet to be defined. For example, a simple measure to evaluate work of the reviewers is to track and analyse the timeliness of the reviewers’ responses, *e.g.* communication with the editor or authors, timely submission of reports before the (soft or hard) deadline, *etc*.

The main components (modules) of DAP are:

- The registration process
- Scholarly Wallet
- Virtual Editor
- Academic Success Ranking
- Open Concurrent Review process
- Advanced Distributed Ledger Technology platform (Tolar HashNET)

### The registration process

For all participants, the interface to the DAP ecosystem is the same – the Scholarly Wallet. The only stakeholder who does not need the Scholarly Wallet is the reader. Since it is an open access publication, any reader can access the entire published work without having to register. However, if a reader whishes to actively comment on and rate the published material, they can only do so through Scholarly Wallet and possibly earn and use DAP Fungible tokens. The DAP ecosystem allows any stakeholder to have multiple roles simultaneously. By choosing different roles, the stakeholder is offered different views of the ecosystem.

The first step for any user is to create a DAP Scholarly Wallet. The wallet generates a public key and a private key based on a random sequence of words. This sequence of words, which is used to generate the private-public key combination, allows the user to access the contents of their wallet independent of certain computer dependencies (a future-proof approach). Once the keys are generated, the party gives the wallet its name and contact information, which is then the first data sent to the blockchain using the Wallet’s public key. This key is the reference identifier of the associated stakeholder. Alternatively, a web-based stakeholder registration template or similar system can be used to download the wallet.

Depending on the role chosen, additional data must now be entered, *e.g.,* areas of interest, keywords, additional descriptions of the stakeholder, *etc.* This is an important part of DAP, as it enables the correct identification of potential reviewers, authors, citations, *etc.* Registration for other roles as well as for other areas of interest and, in general, all changes to the data entered, are made *via* the stakeholder’s Scholarly Wallet.

### Virtual Editor (eEditor)

**Figure 4.**
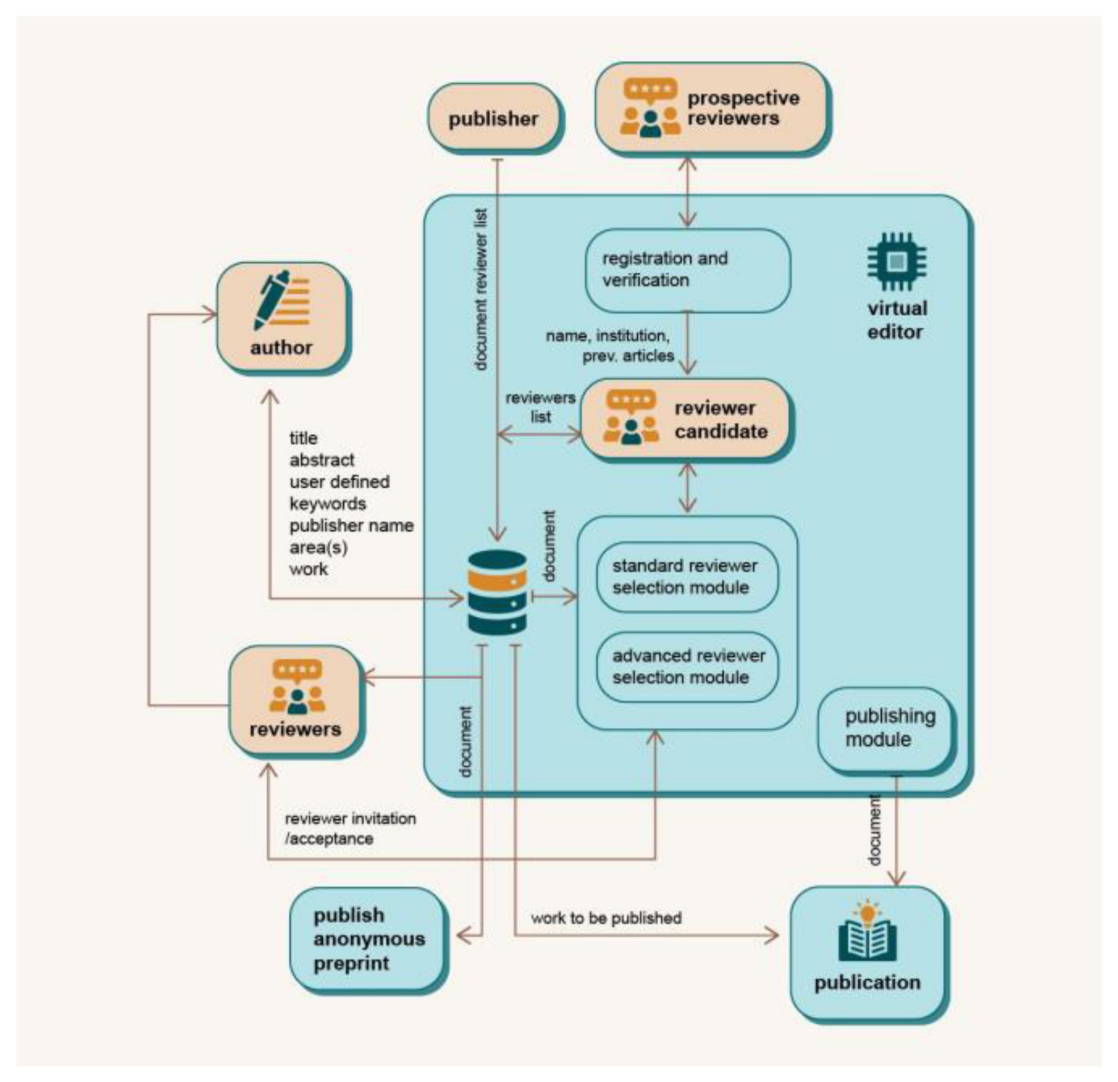
Virtual Editor architecture.

The eEditor module is designed to automate, or at least reduce the work of a human editor by using two modules to select reviewers (see Figure 4). In the standard reviewer selection module, reviewer candidates are selected based on their expertise and ranking, as well as article’s metadata ( *e.g.* research area). The DAP system sends these reviewers an invitation to review the article, and any eligible reviewer can apply for the review. In later stages of development, the advanced module will be used. Using machine learning, the advanced module will enable automatic determination of the research area based on the abstract and content of the manuscript, keywords, *etc.,* allowing more precise and automatic identification of potential reviewers.

During submission, the manuscript is stored to the file system of the DAP server, while the metadata is stored in the blockchain and database. The same process is made available to external publishers. Potential reviewers need to be registered and verified to become candidates for review, or the publisher can add a specific reviewer candidate to the system. The revised and finalised manuscript is published using the publishing module, with the manuscript and metadata stored in relevant databases. In the future, a new, more advanced module could be added to DAP, offering additional features such as the ability to identify the target journal or to check whether the formatting requirements of the respective publisher are met. In this was, authors can ensure that their manuscripts are of the highest quality and ready for publication.

Using a virtual publishing editor like our eEditor alongside a human editor offers several advantages to the publishing process. While eEditor significantly enhances efficiency, it is important to emphasise that human oversight remains essential. eEditor can process a manuscript in a fraction of the time it would take a human editor, reducing the overall time from submission to publication. Additionally, eEditor seamlessly integrates with other publishing platforms, enabling articles to be published directly to websites, databases, or preprint servers. The virtual editing process is not only efficient but also secure, ensuring the privacy and confidentiality of authors’ work while minimising perceived bias. Furthermore, virtual editors assist human editors by providing consistent and reliable preliminary assessments. Leveraging algorithms and machine learning, eEditor helps ensure that editorial standards are met, thereby reducing the risk of errors or inconsistencies and leading to a more streamlined and efficient editorial process. However, it is crucial to note that human editorial oversight remains indispensable in providing nuanced judgment, contextual understanding, and ensuring the highest quality of published work. Human editors bring expertise, critical insight, and a deep understanding of disciplinary norms that complement the efficiency of eEditor, ultimately ensuring the integrity and rigor of the academic publishing process.

### Scholarly Wallet (ScoW)

**Figure 5.**
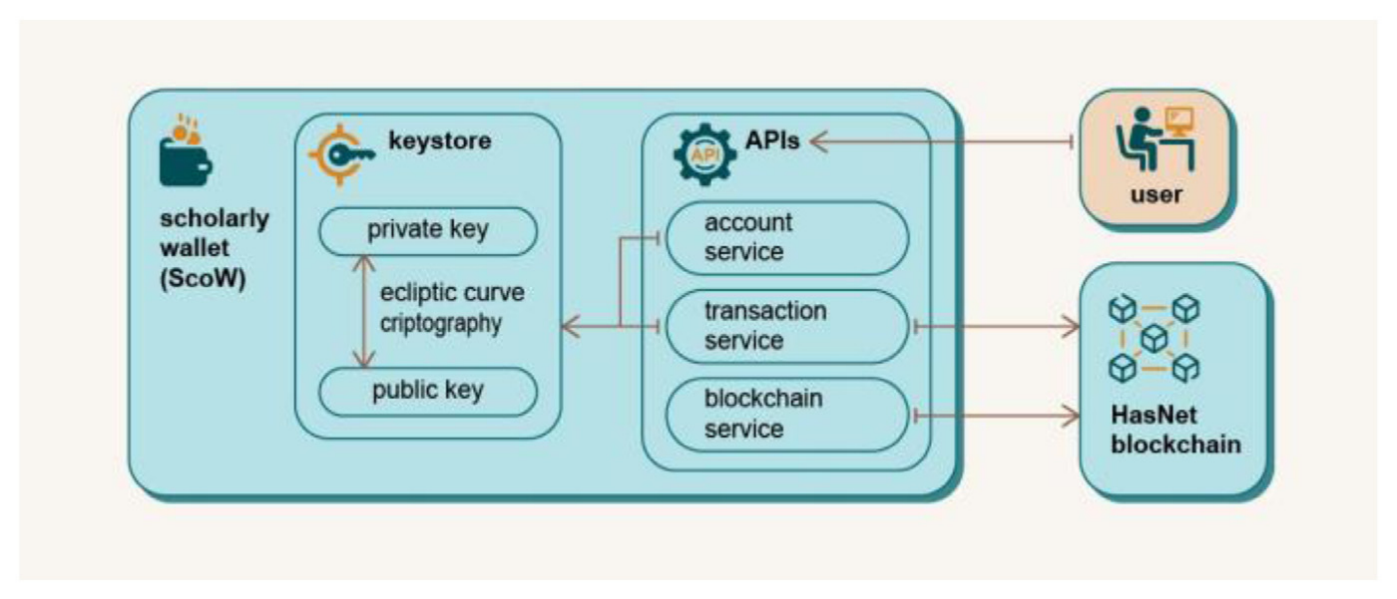
Scholarly Wallet scheme.

The ScoW is software used to interact with the blockchain-based parts of the DAP platform ^25^, and consists of a Keystore and JSON-RPC services. The Keystore is responsible for account management, identification and verification and uses asymmetric cryptography and digital signatures. Elliptic curve cryptography (ECC) was chosen because many other blockchain systems already use the same algorithm, making it compatible with multiple platforms and providing a higher level of security than the classic Rivest–Shamir–Adleman (RSA) algorithm ^26, 27^. The JSON-RPC API uses invocations to distribute data and information between the DAP platform and the blockchain. The ScoW scheme is shown in Figure 5.

When a new public-private key pair is created in the Keystore, the private key is kept secure and the public key (or a derivative of the public key) is used as the identity. It is possible to generate 2 ^256^ of different, completely random private keys. Authors and reviewers can have more than one public-private key pair. This allows them to link their identity, which is essentially the public key, to their real identity or be pseudo-anonymous to blindly publish or review. The wallet is used to send, collect and receive all tokens based on the ERC20 and ERC721 standards associated with user addresses. The wallet uses JSON-RPC API calls to perform all these actions and save changes in the blockchain. By introducing valorisation through the ScoW for participants in the publication process, DAP creates new relationships and opens up the possibility of fair remuneration, increasing the consistency of the scientist. In this way, scientists have functional and operational opportunities to self-finance their scientific activities. The source code of the ScoW is available on Zenodo ^25^.

The introduction of a tradable value, i.e. the token system for reviewing raises concerns about the impact on the intrinsic and extrinsic motivation of reviewers. Placing a tangible value on peer review risks shifting the focus from the intrinsic rewards of contributing to the academic community and advancing knowledge to extrinsic rewards, which could undermine the altruistic nature of peer review. Furthermore, the introduction of a token system could raise questions about fairness and equity among reviewers. If tokens are distributed unequally based on the perceived quality or performance of reviewers, this could lead to comparisons and a sense of unfairness among reviewers. In addition, a token system could unintentionally create a hierarchy among reviewers based on the number or value of tokens received, which could lead to competition and detract from the collaborative spirit of peer review. Reviewers may feel pressured to outperform their peers in order to receive more tokens, which could jeopardise the quality and integrity of the review process. To address this issue, we aim to introduce a range of recognition mechanisms to acknowledge reviewers’ contributions (e.g. public recognition on platforms, certificates of appreciation, invitations to serve on editorial boards or opportunities for professional development), rather than relying solely on token-based incentives. We will ensure transparency and fairness in the allocation of tokens by establishing clear criteria for the distribution of tokens based on the quality and thoroughness of reviews, rather than subjective assessments of reviewers’ performance. The introduction of an objective rating system or peer evaluation process will help maintain fairness and equity among reviewers. The token system will be designed to minimise the potential for creating a hierarchical structure among reviewers by implementing a tiered system that rewards participation rather than performance to prevent competition and comparisons among reviewers. The performance of the peer review process itself will be used as the key to the right of priority in the ranking list for the review of a particular work. The aim is to regularly evaluate the impact of the token system on the motivation of reviewers, their satisfaction and the quality of peer reviews. Feedback from reviewers and stakeholders would also need to be gathered regularly and the token system adjusted based on the results to address any unintended consequences or shortcomings.

### Academic Success Ranking (ASR)

The current standards to measure academic excellence have their pitfalls. The h-index ^28, 29^, for example, does not take into account the total number of citations. If a senior scientist does not publish for a long time, his/her h-index drops to the level of a junior scientist with only a few but highly cited papers, even though a senior scientist has a large number of potentially outstanding papers. The h-index does not take into account wheter the paper was published by only one author, as first or last author, nor what role the author played for the respective paper ( *e.g.* corresponding author). On the other hand, to the best of our knowledge, there are no systems in place to rank, reward and evaluate reviewers at the same time. The closest solution to this may be systems such as the Web of Science Reviewer Recognition Service ^7^, which is designed to reward and verify expert peer reviewers. The results of a study by Lei (2021) ^30^ show that there is no significant difference in the academic impact of reviewers who review papers for journals with a high impact factor and those who are not even indexed in relevant databases.

**Figure 6.**
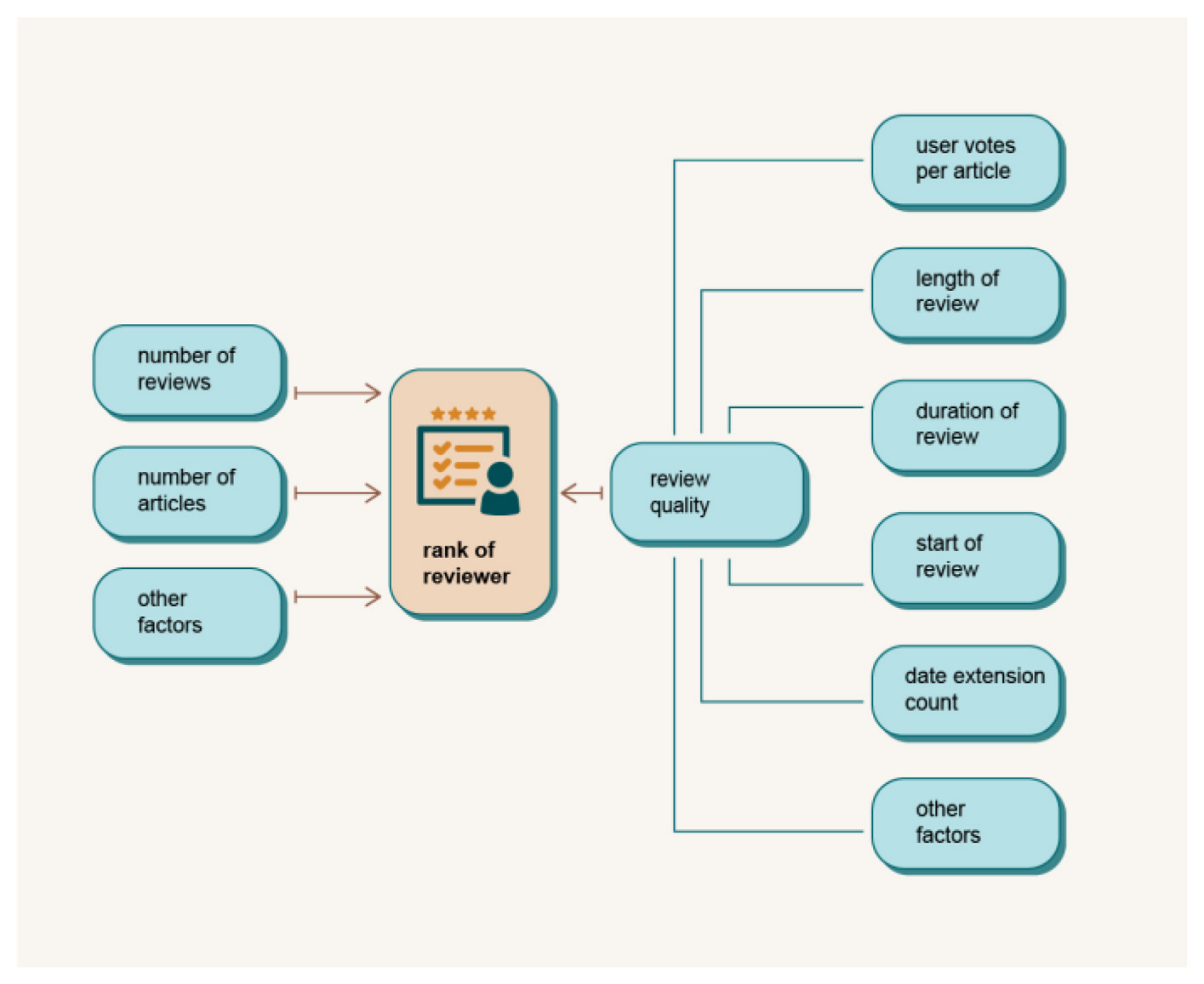
The scheme of the Academic Success Ranking module.

In order to overcome the above mentioned disadvantages of the current systems and to enable a fair ranking of the reviewers, we define the concept and explain the functionality of the ASR module. The module will be build in the implementation phase of the project. The ASR module will evaluate individual efforts in the publication process, especially reviewers, based on the quality of their reports and duration of their effort, *e.g.* the reviewing process. The general rules and criteria of the ranking system will be established during in the implementation phase of the project. A reviewer with a higher rank will have priority in the selection of reviewers for a given manuscript. DAP will make it possible to evaluate not only the quantity but also the quality of reviewers. With a ranking mechanism, authors can be sure that their manuscript will only be reviewed by the best reviewers in the field. ASR provides a fair and transparent way for researchers to be recognised for their contributions, regardless of their affiliation or location. It also helps to build a more accurate and comprehensive picture of an individual’s academic profile, making it easier for potential employers, collaborators and funding bodies. Figure 6 shows some possible elements of reviewer assessment in the ASR module.

### Open Concurrent Review (OCR)

Given that new scientific discoveries are based on proven knowledge and theories, it is of great importance for the academic community to further spread and disseminate the results of studies and make them publicly available to other scientists in the field and beyond. Therefore, science as an institution of organised critique can only develop properly if the results are subjected to the test and scrutiny of other researchers in the field. Peer review is the most important element when it comes to assessing of the quality of a scientific paper. Although it is a well-established process, peer review has also been widely criticised for being too slow and for the editors and reviewers being considered biased. Manuscripts can be held back for several months and progress can be hindered.

**Figure 7.**
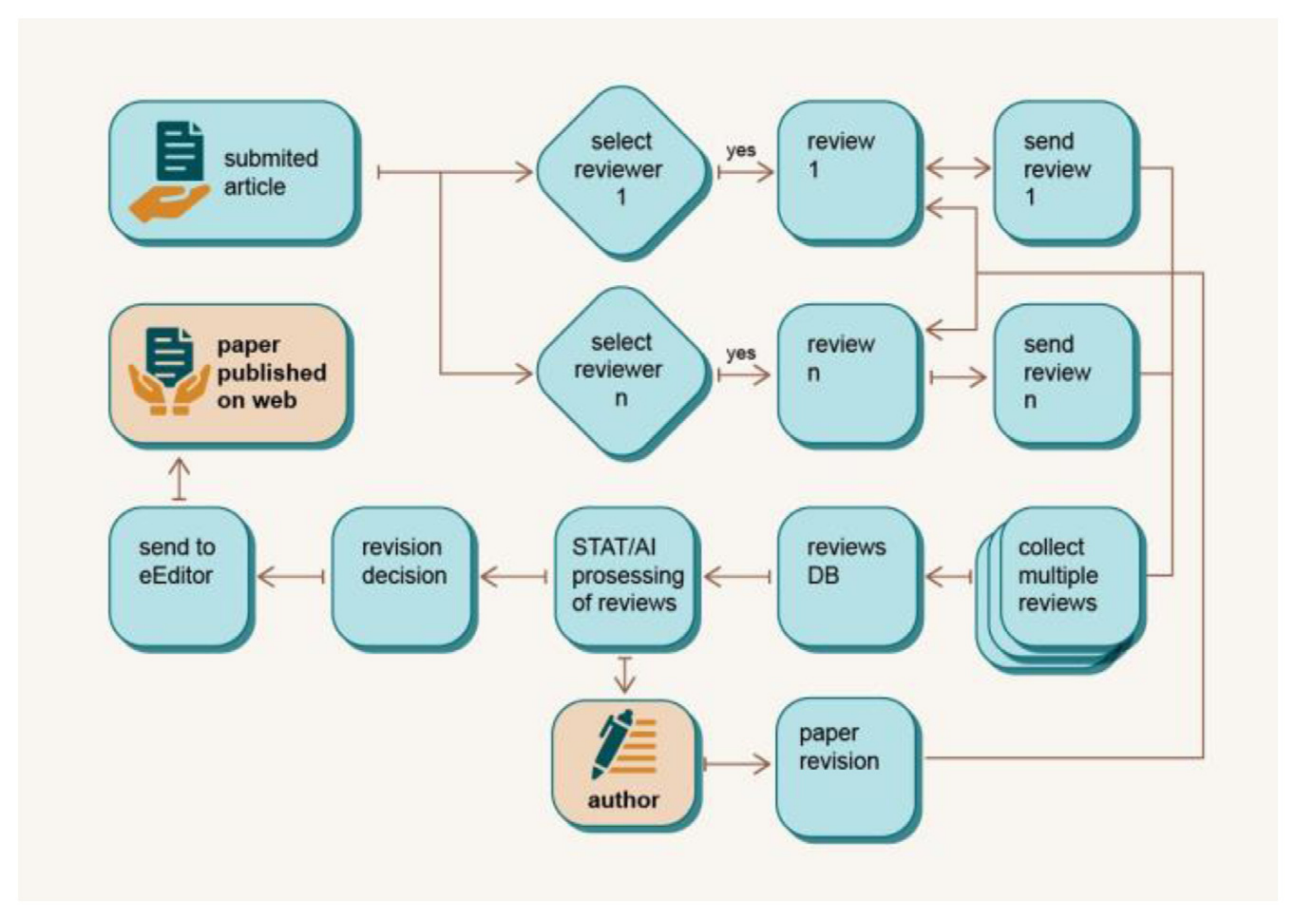
Open Concurrent Review module concept.

In the decentralized OCR model (depicted in Figure 7), the traditional notion of reviewers being individually approached by editors is replaced with a more inclusive and responsive approach. Upon submission, the manuscript is promptly made accessible to a pool of potential reviewers within the reviewer database. This marks the beginning of a decentralized evaluation. As already mentioned, the idea is to divide the tokens equally among the reviewers based on their contribution to the process, i.e. participation in the review. The performance of the reviewers is used to put a reviewer in the vote for reviewing a particular article. The reviewers, who are experts in the field, are invited to review the submitted paper based on their professional interests and expertise. The decentralised nature of OCR gives reviewers the opportunity to decide independently whether to accept or decline the invitation to review. It is important to note that accepting an invitation to review does not mean that every reviewer will participate in the review process, especially for those who are low on the list and have previously submitted poor reviews. Instead, the DAP applies a selection mechanism to ensure a fair and diverse representation of perspectives. From the pool of reviewers who have agreed to participate, an algorithm selects three individuals for the review process who perform best in terms of the quality of a peer review process (and not the quantity). This selection process is not only based on the expertise and suitability of the reviewers, but is also intended to prevent the monopolisation of certain disciplines or topics. By diversifying the pool of reviewers for each submission, DAP ensures a comprehensive and unbiased assessment that reflects the multidimensional nature of scientific research.

Completed reviews are stored in the database and processed with advanced statistical and machine-learning algorithms. In this way, reviews are qualified for further processing in the review process, resulting in a final assessment of the paper. Any revision to the manuscript are processed in the same way, and the final paper is published on the web along with previous versions and the reviewers’ comments. In this way, the review process is accelerated as the editor and/or authors no longer need to search for reviewers as potential reviewers come forward themselves, aiming to increase their ranking and profits. Concurrent and valorised peer reviews not only improve the quality of reviews, but also represent a step forward in the peer review process and the direction in which scholarly publishing is evolving. While the goal is to get more balanced, accurate and faster feedback, multiple reviewers can confuse authors and give conflicting feedback. In such cases, an editor can intervene to resolve the problem. With OCR, the DAP provides a platform for sharing comments, suggestions and feedback in real time, enabling a more efficient and collaborative review process. The key benefit of OCR is that it reduces the time it takes to review and publish, enabling faster dissemination of research results. It also ensures that reviewers are up-to-date with the latest advances in their field, making it easier for them to provide constructive feedback. Voluntary post-publication evaluation to further improve already published papers is not excluded by DAP.

### TOLAR HashNET

To achieve the proposed goals using technology, we need a unique and advanced Distributed Ledger Technology (DLT) platform that contributes to the trustworthiness of published and verified content procedures. This platform must ensure proper and scalable use at the operational level of EU Member States in their holistic global context. The HashNET ^31^ uses the Redundancy Reduced Gossip (RRG) algorithm ^32^ and the Virtual Voting protocol for the transmission of information in an appropriately designed network, resulting in a significantly lower traffic load than when using traditional push or push-pull based gossip protocols. Since less data is transmitted, the processing time can be reduced. The key features of the TOLAR HashNET platform are:

1. **Scalability**: supports up to 20,000 transactions per second on layer 1 and can go into the millions on layer 2 (sidechains). Even with a large increase in the number of nodes, the HashNET network processes all transactions within seconds.
2. **Speed**: It does not require miners to create a chain of blocks to record transactions. HashNET uses a proprietary consensus algorithm to achieve distributed consensus.
3. **Sustainability**: The HashNET platform relies on HashNET’s consensus algorithm, which eliminates the need for massive energy consumption and is, therefore, suitable for public and private services. Virtual voting allows nodes to calculate votes for some nodes instead of sending them over the network, minimising latency and network traffic.

The HashNET algorithm for achieving consensus with the fault tolerance of up to 1/3 of the nodes is described in more detail in 31. To achieve all the functions and goals of DAP, a combination of technologies must be used:

- Private Blockchain based on HashNET is proposed as the blockchain platform,
- SQL Cluster - a high availability database cluster, open source,
- Microservice architecture - application servers to connect all of the components, interface the system,
- Smart contracts - business logic handling on top of the blockchain platform.

## Results

In this section, we describe how the DAP platform will be integrated into the existing digital publishing ecosystem and how it will interact with the research community. Furthermore, following the preliminary results, we give the first insights into the implementation of the Scholarly Wallet module.

### DAP integration

This subsection presents the integration models of the DAP platform into the existing publishing ecosystem. Two main integration paths are envisaged. The first is for DAP to serve as the backend system for tracking scholarly communication, recognising work effort and rewarding all participants of the peer review process and ranking reviewers. The second model envisages DAP acting as a stand-alone publishing platform with its own API interface to the shareholders of DAP, *e.g.,* authors, editors, reviewers and readers.

**Figure 8.**
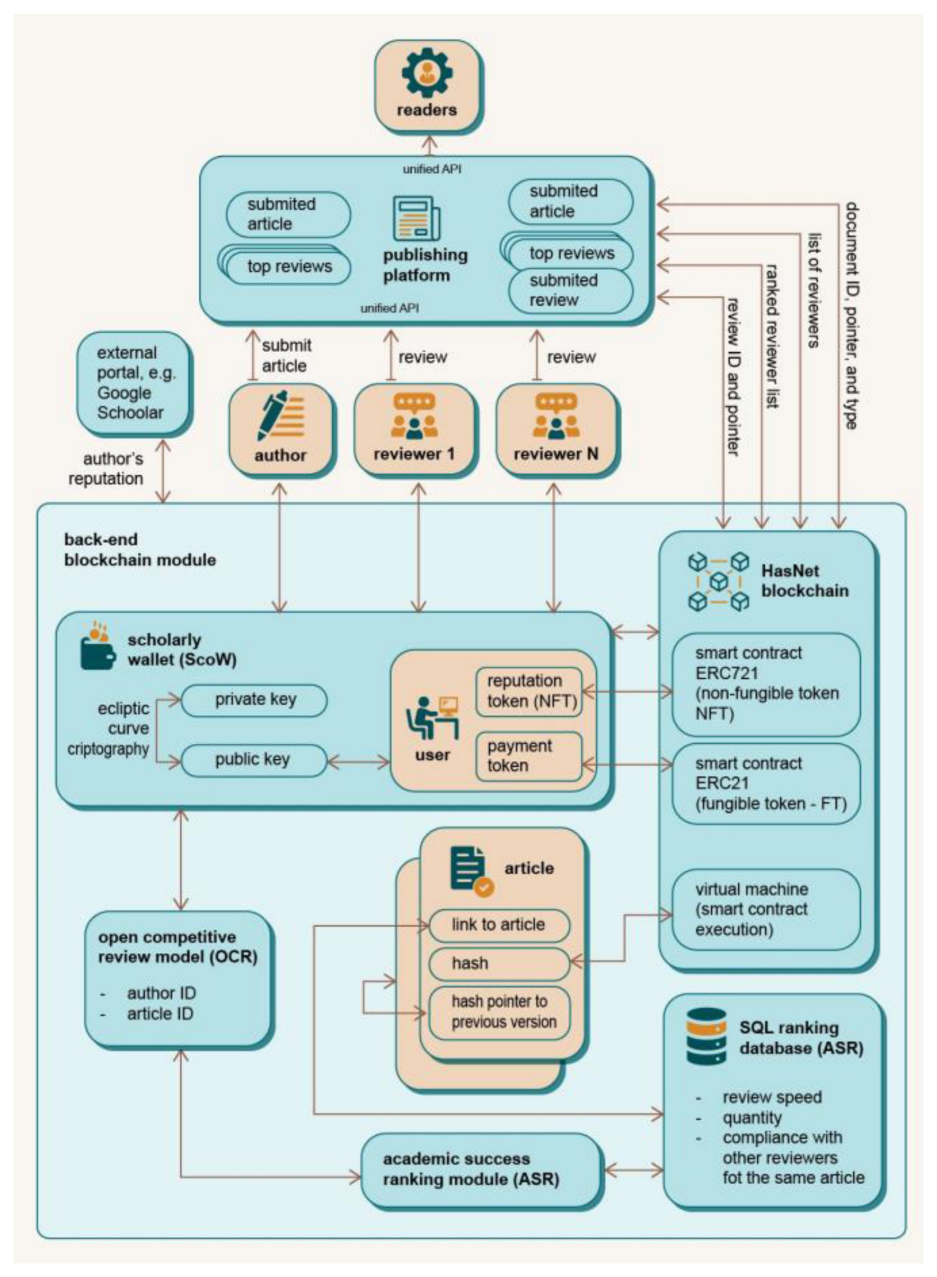
An example of the integration of Decentralised Academic Publishing (DAP) into the existing publisher platforms.

The scenario in Figure 8 represents a block diagram for using the DAP system, where an external publishing platform uses DAP as a backend system. The review process and communication is handled by the publishing platform as before, but all actions ( *e.g.,* an article submitted by authors, a reviewer uploading the report) are tracked directly in the DAP platform (running in the background). The publishing platform must be integrated with ScoW so that the actions performed and the work done can be assigned to the correct participant ( *e.g.,* author or reviewer). The publishing platform must have a unified API to communicate with readers, authors and reviewers. An external publisher must specify the document ID, the pointer and the type of article when forwarding the article’s metadata to the DAP platform. If the publisher wants to submit its reviews, the ReviewID and the pointer to the review must be provided on the publisher’s site.

When users communicate with the DAP blockchain module, they use their ScoW, which stores their reputation and payment tokens. The ScoW address is stored in the backend blockchain module and is used to work with the HashNet blockchain with smart contracts executed by an EVM virtual machine. The Open Competitive Review model requires authorId and articleId for its operation in order to use the Academic Success Ranking module (ASR), which is used to calculate and retrieve all rankings using a SQL ranking database. The SQL Ranking database uses various rating measures to calculate the ratings of an article whose pointer is stored in a file. One of the innovations of the DAP system is that the list of ranked reviewers, who are ranked (ASR) based on their previous work and quality reports submitted, can be suggested to the publisher or editor when deciding which reviewers to invite for each article received.

### Scholarly Wallet

The initial prototype of the Scholarly Wallet is a basic version based on the Tolar HashNET wallet, a high-performance and scalable blockchain platform. It is designed to provide users with a secure and decentralised way to save, manage and transfer the fungible token that is the central component of the DAP system. The wallet allows users to generate HD keys, import and export keys and send and receive fungible ERC20 tokens while connecting to the HashNET-based DLT.

The wallet is built as a web3-based browser plugin that allows users to interact with web-based CMS (Content Management System) front-ends without having to run a full blockchain node. This allows for a seamless user experience as users can access and manage their tokens directly from their browsers.

An ERC20-based Ergion token has been developed and deployed on the CroBSI ^8^ test network to be used within the academic community as a means of rewarding authors, reviewers, and other members of the ecosystem. The Ergion token can be used as an incentive for high-quality research and scholarly content, as well as to reward those who contribute to the peer review process.

For the sake of simplicity, but to keep the manuscript self-consistent, we briefly describe below how to install ScoW on a local computer as a plugin for the Chrome web browser. Detailed instructions on how to download and install Scholarly Wallet can be found in 25 (see *Installation.pdf*).

**Figure 9.**
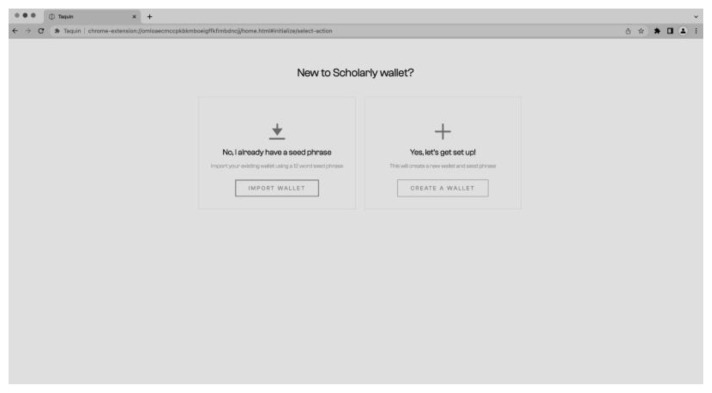
Import of existing or creating a new wallet.

**Figure 10.**
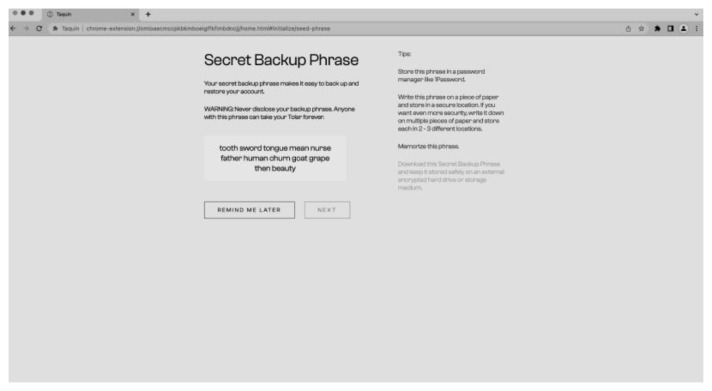
New seed phrase for the wallet.

The process of creating the wallet and connecting to the CMS is straightforward and user-friendly. The user has the option to import existing keys or a seed phrase if they already have a wallet or can create a completely new wallet (see Figure 9) by generating a seed phrase and setting a password ( Figure 10). The process of creating a new wallet involves generating a seed phrase which acts as a backup and recovery mechanism for the wallet. The user is also prompted to set a password to secure the wallet. This combination of seed phrase and password ensures security and access to the user’s assets stored in the wallet.

**Figure 11.**
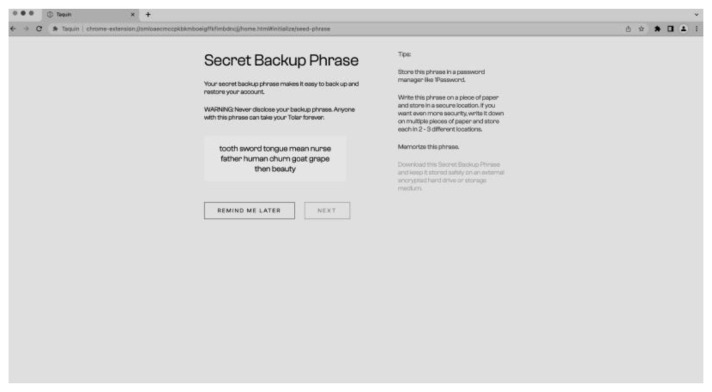
Scholarly Wallet with 33 Ergions.

Next, the user can connect to the CMS by providing their wallet address and connecting to the CroBSI test network. Once connected, the user can receive Ergion tokens and send them to other users within the academic community. The wallet also displays the user’s current account balance and transaction history, making it easy to track and manage their Ergion tokens as presented in Figure 11.

**Figure 12.**
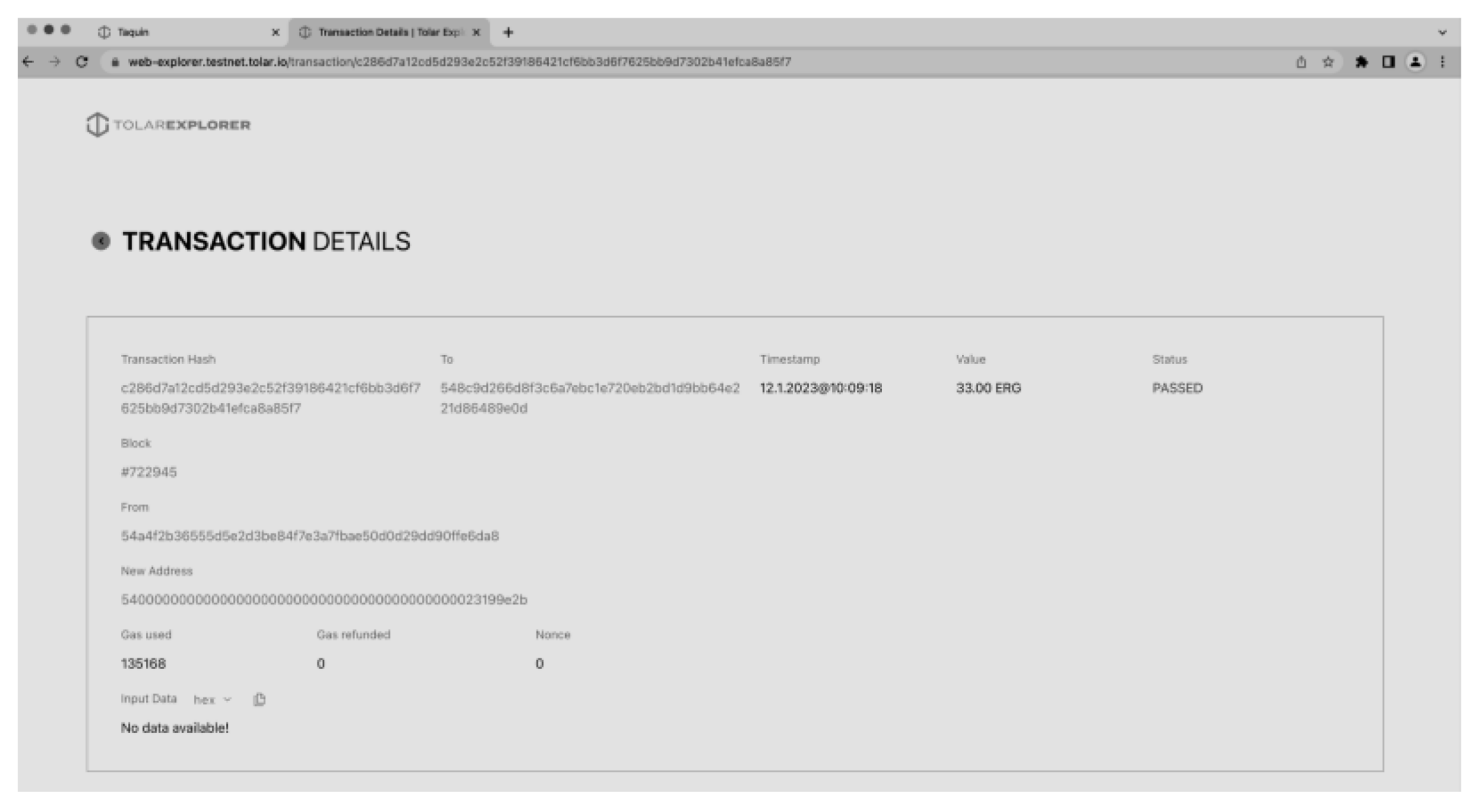
Transaction information on HashNET explorer.

In Figure 12 one can see an example of a transaction of 33 ERG from one wallet to another. The process of transferring Ergion tokens from one wallet to another is a fundamental aspect of the Scholarly Wallet and is based on the decentralised and trustless nature of the HashNET blockchain. The transaction process begins with the sender initiating the transfer by entering the recipient’s wallet address and the amount of Ergion tokens to be sent. The sender must also confirm the transaction by signing it with their private key, which is securely stored in the Scholarly Wallet.

Overall, the initial prototype of the Scholarly Wallet combined with the Ergion Token represents an important step towards creating a decentralised and incentivised ecosystem for the academic community. It is expected that the use of blockchain technology and the Ergion token will lead to a more efficient and transparent way of rewarding authors and reviewers, which will ultimately advance scientific research.

### ScoW testing

The Scholarly Wallet was tested on the HashNET test network to verify its functionality in both simulated and real-world scenarios. The test procedure is described in more detail in 25 (see *TESTING.pdf*, section "Manual testing").

The test platforms consisted of the PCs with the macOS 13, Ubuntu 20.04 LTS and Windows 10 operating systems, the Chrome web browser version 114 and a stable internet connection. The ScoW version *1.0.2* was downloaded and installed locally. The test blockchain network was the HashNET test network. Detailed instructions on how to connect to the HashNET blockchain test network can be found in the installation guide ^9^ (see 25).

The focus of the testing was on validating the correct behaviour of the ScoW plugin application and its interaction with the HashNET blockchain network. Four key functionalities of the ScoW were tested each of which is said to be correct if the above mentioned steps can be conducted without errors.

**Installation and configuration:** The wallet was downloaded and installed on a clean computer, following the installation instructions in 25 in *INSTALLATION.pdf*. The configuration process was then carried out, including the generation of a HD key and the import of an existing key. For testing, the “Testnet” network was used.

**Key management:** Key management was tested, including exporting a HD key and deleting an imported key. The tests were carried out as follows. A new wallet was created according to the instructions described above and in 25. A new seed phrase was created when the wallet was created. The seed phrase is stored locally or written down. Within the new wallet, five new addresses (accounts) were created, each with its own key pair (public and private). The wallet (plugin) is then completely removed from the browser and deleted from the computer. Then the ScoW plugin is installed on a new computer and a new wallet is created, but instead of creating a new wallet (and generating a new seed phrase), this time the existing wallet is imported, *i.e.* the previously generated seed phrase (see 25 *TESTING.pds* subsection “Create a New Wallet”). The validity of the wallet is confirmed when the same addresses (accounts) are created with exactly the same key pairs, the token balance is maintained and one can perform transactions. The test wallet and its accounts were successfully recreated, new transactions executed and their status confirmed in the Transaction Explorer.

**Token management:** The sending and receiving of ERC20 tokens has been tested, including the creation of a token transaction, the display of the transaction history and the receipt of tokens. The test tokens are requested *via* the query specifically set up for testing purposes and only available on the HashNET network “Testnet”. For detailed instructions on how to register with Testnet and request test tokens, see 25 in *TESTING.pdf* (subsection "Add test tokens to new address").

The token transaction mechanism was tested by executing several hundred transfers between multiple accounts (addresses) within multiple wallets on multiple computers. How to transfer tokens between accounts is described in 25 (document *TESTING.pdf* section “Testing token transfers”). The validity of the executed transaction can be monitored and confirmed using the Testnet Transaction Explorer monitor. For more information check ^25^ (document *TESTING.pdf* section “4. Check transaction on explorer”).

**Security:** The security of the wallet was tested, including the strength of encryption, protection against phishing attacks, and the ability to recover lost keys. You can test the strength of the encryption by trying to enter the ScoW without a password. It is recommended to set a strong password when creating the wallet. In this case, even the brute force method requires a lot of time and computing power to crack it. In addition, the wallet contains a list of possible phishing websites, which are then automatically rejected. To test the recovery of the lost keys, you can recreate the wallet with the seed phrase. In the previous tests ( *key management*) we have confirmed that the accounts, keys and token balances are preserved and can also be easily restored on a new computer (browser).

Although the testing was successful, the wallet is still in the pilot phase and needs further development until it is ready for production. The implementation of the fully functional DAP platform is planned in the HorizonEurope Trace4EU project and other research initiatives. Adding support for non-fungible tokens (NFTs) and other token standards would make the wallet even more versatile. The NFT tokens will be used as reputation tokens for the ranking of the participants.

## Conclusions

The paper "Prospects of digital scientific publishing on blockchain" proposes the use of Decentralised Academic Publishing (DAP) to improve the current system of scientific publishing by making it more transparent, secure and traceable. DAP will use blockchain technology to make research metadata read-only, secure and more visible. It will also use scientific tokens (Ergion) to reward reviewers, editors and authors. By using blockchain technology, DAP can offer faster and more efficient publication processes, increase accessibility and reduce the potential for fraud. DAP will be a decentralised system managed by a community of users rather than a central authority and will use smart contracts to automate processes such as peer review services and increase transparency. Overall, the authors believe that DAP has the potential to significantly improve scientific peer review and knowledge dissemination.

In conclusion, the use of blockchain technology in scientific publishing offers many promising benefits. Blockchain-based systems can provide secure and tamper-proof ways to store and share scientific data, increasing transparency, accountability, and reproducibility in the scientific community. By eliminating intermediaries and enabling direct interactions between authors, reviewers and readers, blockchain-based platforms can also reduce costs and enhance the speed and efficiency of scientific publishing.

The potential benefits of blockchain-based systems in scientific publishing make them a promising area for future research and development. As more researchers and publishers become familiar with the technology and its advantages, we can expect to see an increase in the use of blockchain-based systems for scientific publishing in the coming years. Overall, the prospects of digital scientific publishing on blockchain are promising, and the technology has the potential to transform the way we publish and share scientific knowledge.

## Ethics and consent

Ethical approval and consent were not required.

## Data Availability

No data associated with this article.

## Software availability

Archived source code at the time of publication: https://doi.org/10.5281/zenodo.8034931 ^25^

License: MIT
