# Supplementary material for: Prospects of digital scientific publishing on blockchain: The concept of DAP
Source: Open Res Eur. 2024 Nov 27;3:117. Originally published 2023 Jul 20. [Version 2] doi: 10.12688/openreseurope.15771.2 (PMC11809477; doi:10.12688/openreseurope.15771.2)
Supplement: Peer review history

# Reviewer report: Reviewer response for version 2

**DOI:** 10.21956/openreseurope.19525.r47631

**Published:** 2024-12-30

**Authors:** Sandip Majumdar

**Affiliations:** 1 University of Gour Banga, Malda, West Bengal, India

I have gone through the article. My observations are as follows:

 1. I doubt the clarity of the title of the manuscript as the "Prospects of digital scientific publishing on Blockchain..." may confusingly mean articles on digital scientific publishing on the subject Blockchain. An alternative title may be considered as "Prospects of digital scientific publishing based on blockchain: The concept of DAP".

 2. The authors mentioned the need for increased women participation in the peer review process but have not demonstrated explicitly in their DAP system the mechanism to do so.  

 3. The whole process of evaluating works of peers seems somewhat mechanical as nothing has been mentioned about the assessment of intellectual content of the review reports for their scholarship and associated sentiment analysis for detection of praise and criticism in peer evaluation. In this respect application of Artificial Intelligence (AI) may enhance assessing the intellectual content of a review report. In the age of AI systems without application of AI may seem backdated. Authors may go through experimentation with PeerJudge (refer 1) for nuanced understanding of integration of AI in evaluation of intellectual content of peer review report for assessing worthiness of a reviewer for future review.

Is the rationale for developing the new method (or application) clearly explained?

Partly

Is the description of the method technically sound?

Yes

Are the conclusions about the method and its performance adequately supported by the findings presented in the article?

Yes

If any results are presented, are all the source data underlying the results available to ensure full reproducibility?

Yes

Are sufficient details provided to allow replication of the method development and its use by others?

Yes

Reviewer Expertise:

Scholarly communication

I confirm that I have read this submission and believe that I have an appropriate level of expertise to confirm that it is of an acceptable scientific standard.

## References

[1] : Automatically detecting open academic review praise and criticism. *Online Information Review* .2020;44(5) : 10.1108/OIR-11-2019-0347 1057-1076 10.1108/OIR-11-2019-0347

# Reviewer report: Reviewer response for version 2

**DOI:** 10.21956/openreseurope.19525.r47634

**Published:** 2024-12-27

**Authors:** Zihan Zhou

**Affiliations:** 1 The Hong Kong University of Science and Technology, Guangzhou, China

The article presents a well-conceived method for improving academic publishing through blockchain technology, specifically through the DAP platform. The rationale for developing this new method is clearly explained, and the technical description of the method is sound. Sufficient details are provided to allow replication of the method development and its use by others. However, the conclusions about the method and its performance are only partly supported by the findings presented.

 The primary concern lies in the lack of empirical evidence to substantiate the effectiveness of the DAP platform in motivating reviewers to leave existing platforms. The article does not include any experiments or surveys to demonstrate the actual motivating power of the proposed platform. To address this, the authors should consider conducting pilot studies or surveys to gather data on reviewer preferences and motivations regarding the use of the platform.

 In addition, the article describes the use of smart contracts for file access control but does not delve into the specifics of file encryption, decryption, or the location of file storage. It would be beneficial to provide more detailed information on these aspects to ensure the integrity and confidentiality of the published research.

 Furthermore, the authors could explore more in using AI. You might find inspiration in the article “A Blockchain-enabled Peer Review Platform” (arXiv:2405.20220), which utilizes LLM to generate summaries. Building upon this, the authors could consider integrating AI as a peer review entity, serving as a virtual editor.

Is the rationale for developing the new method (or application) clearly explained?

Yes

Is the description of the method technically sound?

Yes

Are the conclusions about the method and its performance adequately supported by the findings presented in the article?

Partly

If any results are presented, are all the source data underlying the results available to ensure full reproducibility?

Yes

Are sufficient details provided to allow replication of the method development and its use by others?

Yes

Reviewer Expertise:

Blockchain, Decentralized AI

I confirm that I have read this submission and believe that I have an appropriate level of expertise to confirm that it is of an acceptable scientific standard, however I have significant reservations, as outlined above.

# Reviewer report: Reviewer response for version 2

**DOI:** 10.21956/openreseurope.19525.r41567

**Published:** 2024-12-17

**Authors:** Teodor Mihaela

**Affiliations:** 1 “Mihai Viteazul” National Intelligence Academy, București, Romania

No further comments to make

Is the rationale for developing the new method (or application) clearly explained?

Yes

Is the description of the method technically sound?

Yes

Are the conclusions about the method and its performance adequately supported by the findings presented in the article?

Partly

If any results are presented, are all the source data underlying the results available to ensure full reproducibility?

No source data required

Are sufficient details provided to allow replication of the method development and its use by others?

Yes

Reviewer Expertise:

Researcher in history of media and communication; Reviewer and editor of scientific publications; Interested in AI use for publishing and editorial processes

I confirm that I have read this submission and believe that I have an appropriate level of expertise to confirm that it is of an acceptable scientific standard.

# Reviewer report: Reviewer response for version 2

**DOI:** 10.21956/openreseurope.19525.r47560

**Published:** 2024-12-13

**Authors:** Sara Kärrholm

**Affiliations:** 1 Associate Professor of Literary Studies, Division of Publishing Studies, Lund University, Lund, Sweden

The article has been revised with thoroughness and meet most of the requests made in the peer review report provided for the first version. Several of the arguments and suggestions from the authors have been nuanced and problematized in a manner that I find is sufficient. The language is now clear and the concepts used and the process of the investigation is described in a more pedagogical way than previously. 

 I suggest that one inconsistency should be addressed, though, which is the mentioning of DAP as Democratisation of Academic Publishing in the abstract, since the article text uses the wording of DAP as Decentralized Academic Publishing.

 In other words, I conclude that this article is ready for indexing with one minor revision.

Is the rationale for developing the new method (or application) clearly explained?

Partly

Is the description of the method technically sound?

Yes

Are the conclusions about the method and its performance adequately supported by the findings presented in the article?

Partly

If any results are presented, are all the source data underlying the results available to ensure full reproducibility?

No source data required

Are sufficient details provided to allow replication of the method development and its use by others?

Yes

Reviewer Expertise:

Publishing, literature, digital cultures. I am approaching this article from a viewpoint of a humanities scholar, which makes me less suitable to comment on the parts of the article that are related to computer science.

I confirm that I have read this submission and believe that I have an appropriate level of expertise to confirm that it is of an acceptable scientific standard.

# Reviewer report: Reviewer response for version 1

**DOI:** 10.21956/openreseurope.17036.r41401

**Published:** 2024-06-27

**Authors:** Teodor Mihaela

**Affiliations:** 1 “Mihai Viteazul” National Intelligence Academy, București, Romania

The authors identify the gaps, needs and challenges of the current academic editorial and publishing processes and propose both a new method and application to address the speed of peer review, the recognition of work and the incentive mechanisms, the transparency and thrust of the system. They propose the use of blockchain technology for the development of the Decentralised Academic Publishing (DAP) platform in order to address the challenges identified and to reach a more efficient and transparent publishing ecosystem including a way of rewarding all participants in the peer review process. The system uses innovative elements such as a virtual editorial system, an open and competitive peer review process, the Ergion token and the Scholarly Wallet (ScoW) as incentive mechanisms.

  

1. The rationale for the new platform is clearly explained. The authors try to address the key challenges of academic publishing today: speed of peer review, recognition of work and incentive mechanisms, transparency and thrust of the system. However, the introductory part misses a clear statement on the funding project and the connection of the paper with this project which could give us a better understanding of the context and the background of the new method/ application proposed.
2. The description of the method is correct and detailed from a technical perspective being the fact that the paper is more about the presentation of the DAP design and functionalities with its central components: Scholarly Wallet, Ergion token and eEditor module.  
3. There are some gaps when the authors try to explain the proposed recognition of work and incentive mechanisms for reviewers’ work. The DAP platform aims to rank, reward and evaluate reviewers at the same time. However, it is not clear how the reviewers would be evaluate and ranked? They would be `ranked by the quality of their previous work in the review process` or `the ranking criteria would be established in the next phase of the platform development – the ASR module`. Would the criteria be quantitative and qualitative?
4. The Scholarly Wallet (ScoW) and the digital currency is already in the implementation phase, as the authors stated. Which is the impact and the rate of acceptance? What are the disadvantages of using a virtual publishing editor instead of a human editor?
5. The concluding remarks are based on a single idea that `the use of blockchain technology in scientific publishing offers many promising benefits`. However, could there exist any limitations and negative aspects? Could the connection of DAP to present publishing ecosystem be better explained? How it could be accepted and implemented, only on a voluntarily basis?
6. There are some inconsistencies in using the proposed concept DAP:  Decentralised Academic Publishing and Democratisation of Academic Publishing. Could authors explain in the introductory part the link between the decentralization and democratization at a conceptual level, or they could use only the term Decentralised for DAP platform, the decentralization being the central idea of their technical solution as the authors highlighted in the paper.  

Is the rationale for developing the new method (or application) clearly explained?

Yes

Is the description of the method technically sound?

Yes

Are the conclusions about the method and its performance adequately supported by the findings presented in the article?

Partly

If any results are presented, are all the source data underlying the results available to ensure full reproducibility?

No source data required

Are sufficient details provided to allow replication of the method development and its use by others?

Yes

Reviewer Expertise:

Researcher in history of media and communication; Reviewer and editor of scientific publications; Interested in AI use for publishing and editorial processes

I confirm that I have read this submission and believe that I have an appropriate level of expertise to confirm that it is of an acceptable scientific standard, however I have significant reservations, as outlined above.

# Reviewer report: Reviewer response for version 1

**DOI:** 10.21956/openreseurope.17036.r35627

**Published:** 2023-11-02

**Authors:** Nicola Cavalli

**Affiliations:** 1 Università di Milano Bicocca, Milan, Italy

The article addresses the issues with peer review in aspect such as the difficulty in finding the right peer reviewer, and in ensuring the quality of the review reports. It seems also to address Open Access Publishing, but with less details.

 The peer review process has been since long time in question in the scientific community, and many proposals have arisen during the years. The article proposed to solve using blockchain technology, applied to academic publishing. t's not clear whether it proposes to introduce new impact metrics, but it seems to, even if that part is only briefly considered. The blockchain use is called DAP (Decentralised Academic Publishing through the implementation of a “Scholarly Wallet”, a system where academic work, such as peer review, is rewarded with 'Ergion Tokens', a digital currency. The proposed platform and its functions are described in detail with elaborate flux models, that are clear enough.

1. The general idea of creating a distributed and shared system for different platforms is interesting but the discontent regarding current peer review needs further investigation.
2. The proposal is weak in general terms as it does not address the core issue of quality definition and solves the definition of quality with just a few quantitative metrics. It is more a general description of how a distributed system based on blockchain might be used to optimize peer review process, but the core problem with peer review is not the process in itself, which of course can be optimized using new technologies, but rather is how the quality of the outputs can be improved.
3. The article is solid in the technical design of the system and sounds feasible, while it is not clear whether it would be desirable or not and the question is not addressed properly.
4. In the introduction there’s a tentative definition of various Open Access models, but it’s not clear in itself and it’s not clear while Open Access is relevant here, as peer review occurs both in closed and open publishing.

Is the rationale for developing the new method (or application) clearly explained?

Yes

Is the description of the method technically sound?

Yes

Are the conclusions about the method and its performance adequately supported by the findings presented in the article?

Partly

If any results are presented, are all the source data underlying the results available to ensure full reproducibility?

No source data required

Are sufficient details provided to allow replication of the method development and its use by others?

Partly

Reviewer Expertise:

Academic publishing, educational technologies, media studies

I confirm that I have read this submission and believe that I have an appropriate level of expertise to confirm that it is of an acceptable scientific standard, however I have significant reservations, as outlined above.

# Reviewer report: Reviewer response for version 1

**DOI:** 10.21956/openreseurope.17036.r35299

**Published:** 2023-11-01

**Authors:** Sara Kärrholm

**Affiliations:** 1 Associate Professor of Literary Studies, Division of Publishing Studies, Lund University, Lund, Sweden

This article describes in detail some perceived problematics with open access publications and the peer review-process, mostly centered on the latter and on the difficulties of both finding the right peer reviewers for articles, how to properly award them, and how to ensure a high quality of the review reports. Many of the issues raised are well known and often addressed within the research area of academic publishing. The article goes on to propose a Decentralised Academic Publishing-platform (later in the article called Democratisation of Academic publishing), DAP. The main way to solve the described problems is suggested as the Scholarly Wallet, a system where academic work, such as peer reviewing, is rewarded with "Ergion tokens", a digital currency. The proposed platform and its functions are described in detail and with elaborate visual models, in a pedagogical way. However, the proposed solutions, in my view, raise some concerns.

1. The idea of monetizing the peer review process is questionable for several reasons, especially if you want to present the platform as driven by democratic ideals rather than commercial. There are no, ethical or other, considerations regarding the consequences of this presented in the article. There are scientific contributions to the discussion about the funding of academic publishing and review process that might be interesting for the writers to address ^1^.
2. The DAP-platform is described as Decentralised Academic Publishing in the abstract, but as Democratisation of Academic Publishing in the text. There is no convincing argument made about why this platform would be more democratic than others, and no explanation of what democracy means in this context. At page 7, the DAP ecosystem is described as a virtual place where different partakers have the right to "vote", but there is no explanation of what this voting concerns or why voting will take place.
3. Another problematic issue is the suggestion that a virtual editor would be "higly consistent and reliable" (p. 10). I can agree on the suggestion that an eEditor might be free from bias and time efficient, but to my knowledge a real editor is the only way to really ensure that someone has a comprehensive view of the article and the review process, while machines are likely to make errors due to a lack of this. Maybe this is an issue that is less problematic in research fields that are concerning themselves with data and numbers, rather than hermeneutic considerations and arguments, as in the Humanities, where I am myself based. It is however an issue worth some problematizing in this context. 
4. Considering that the proposed system is designed to be able to evaluate the work of reviewers, the writers should provide a more thorough list of what makes up the qualities of good reviewing. So far, they concentrate on the timeliness of the review process, which is one aspect, but in my view not necessarily the most important. If reviewers are to build up their reputations and also make money on their reviews, I find that just concentrating on timeliness might undermine the quality of the reviews.
5. Overall, the argument of the article reads very much as and advertisement of the proposed DAP-system. For it to gain substance and scientific credibility, it needs to be presented in a more nuanced way, that takes in the possible limitations to this system as well as its potential.
6. The article should also be checked for spelling, since it is not always clear on the language level.

Is the rationale for developing the new method (or application) clearly explained?

Partly

Is the description of the method technically sound?

Yes

Are the conclusions about the method and its performance adequately supported by the findings presented in the article?

Partly

If any results are presented, are all the source data underlying the results available to ensure full reproducibility?

No source data required

Are sufficient details provided to allow replication of the method development and its use by others?

Yes

Reviewer Expertise:

Publishing, literature, digital cultures. I am approaching this article from a viewpoint of a humanities scholar, which makes me less suitable to comment on the parts of the article that are related to computer science.

I confirm that I have read this submission and believe that I have an appropriate level of expertise to state that I do not consider it to be of an acceptable scientific standard, for reasons outlined above.

## References

[1] : Replacing academic journals. *Royal Society Open Science* .2023;10(7) : 10.1098/rsos.230206 10.1098/rsos.230206 PMC1072308438107166

# Reviewer report: Reviewer response for version 1

**DOI:** 10.21956/openreseurope.17036.r34389

**Published:** 2023-08-25

**Authors:** Eric A. Fong

**Affiliations:** 1 The University of Alabama in Huntsville, Huntsville, Alabama, USA

This paper provides an alternative to the traditional publication process in terms of editors finding reviewers, reviewers being recognized for their work, etc.  The alternative approach provided here seems to monetize the review process to some extent by creating a blockchain “token” that reviewers can earn by reviewing. However, I’m really concerned with the idea that reviewing should be monetized or rewarded in such a way. Of course, this changes the dynamics of reviewing, which is what the authors intend; it likely solves some issues, but raises others. A central concern, really, is the fact that this paper is just supposition; here is what we think is the problem and here is how we think it should be resolved. Okay, what can a reviewer say to that? I agree or disagree. There isn’t any theory development or hypothesis testing. Sure, blockchain can be used this way, but I’m not convinced it should. Moreover, the paper only focuses on the benefits to doing this, what are the costs? Here are my comments and concerns:

1. Although I see that the authors have addressed the idea that academics think their institutions should better recognize their reviewing work, what I find interesting is that the Publons article cited in the manuscript found that 70.6% of respondents stated that when they declined to review an article it was because the “Article was outside my area of expertise.” Is it really that reviewers aren’t being appreciated or rewarded? Or, is it that now there are so many journals, including open publishers, with editors who just need someone to review a paper that they will pretty much send the paper to anyone who has research tangentially related to the topic? Really, is making a digital coin going to solve this problem? If this coin becomes valuable, then people might just go ahead and review even if the paper isn’t exactly in their area of expertise just to get the asset. Is that efficient and/or effective?
2. Changing the norms around why academics should review can have significant negative impacts. Yes, there are some really greedy people out there who likely view the world as wanting to get something for reviewing (even though they expect other people to volunteer to review their papers); however, I want to believe that most academics are professionals and do their best to give back to the academy as well as move forward science. This, of course, is testable. When we start to put some type of tradable value on reviewing, this becomes a problem (we could be impacting Intrinsic/Extrinsic motivation; this is based on theory). Moreover, will this system treat all reviewers the same? If you are a great reviewer and I’m only a good reviewer, do you get more tokens for reviewing? If we get the same number of tokens or if we get a different number of tokens, comparisons suddenly start coming into play, that really starts to change people’s motivation in problematic ways (Equity Theory starts coming into play).
3. In the section of “Open Concurrent Review” it seems the authors are describing the process as “potential reviewers come forward themselves, aiming to increase their ranking and profits.” I really am struggling with the prospect of “gamifying” and paying for the review process. Is it possible that whole parts of disciplines/topics become cornered off because a small set of reviewers “volunteer” for papers on particular topics; in turn, crowding out other views?
4. The introduction seems out of place. I’m not sure why knowing the difference between academic publishing models matters. Is DAP being introduced for only one model; the OA model? The traditional publishing model would continue on as is? I think an introduction that better fits the paper is necessary. The reader doesn’t really get what the authors are trying to accomplish in this introduction.

Is the rationale for developing the new method (or application) clearly explained?

Partly

Is the description of the method technically sound?

Yes

Are the conclusions about the method and its performance adequately supported by the findings presented in the article?

Partly

If any results are presented, are all the source data underlying the results available to ensure full reproducibility?

No source data required

Are sufficient details provided to allow replication of the method development and its use by others?

Yes

Reviewer Expertise:

Academic/Research integrity, Firm Governance, CEO Compensation, Ecosystems, Innovation

I confirm that I have read this submission and believe that I have an appropriate level of expertise to state that I do not consider it to be of an acceptable scientific standard, for reasons outlined above.
